# Simultaneous integrated boost intensity-modulated radiotherapy for locally advanced non-small cell lung cancer in Chinese population: A retrospective study

**DOI:** 10.18632/oncotarget.17094

**Published:** 2017-04-13

**Authors:** Yujin Xu, Xiao Zheng, Xue Bai, Pu Li, Honglian Ma, Jin Wang, Xiao Hu, Ming Chen

**Affiliations:** ^1^ Department of Radiation Oncology, Zhejiang Cancer Hospital, Hangzhou, 310022, China; ^2^ Zhejiang Provincial Key Laboratory of Radiation Oncology, Hangzhou, 310022, China; ^3^ Department of Radiation Physics, Zhejiang Cancer Hospital, Hangzhou, 310022, China

**Keywords:** non-small cell lung cancer, integrated boost intensity-modulated radiotherapy, local control, overall survival

## Abstract

**Objectives:**

To evaluate the clinical efficacy and toxicity of simultaneous integrated boost intensity-modulated radiotherapy (SIB-IMRT) for patients with locally advanced non-small cell lung cancer (NSCLC).

**Results:**

All patients completed definitive radiotherapy and 74 (85.1%) patients administrated platinum-based chemotherapy. The median radiation dose was 50.4Gy to PTV and 64.4 Gy simultaneously to the PGTV. The overall response rate (ORR) was 57.5% (50/87). The median duration of follow up was 24.6 months. The 1, 2, 3-year local control rate was 79.0%, 66.1%, and 60.5%, respectively. The 1, 2, 3-year overall survival (OS) rate was 89.7%, 56.7%, and 30.6%, respectively. Subgroup analysis showed that the median OS in concurrent chemoradiation (CCRT) was much better than non-CCRT (35.7 vs. 26.4 months) (HR: 0.52, 95% CI: 0.32–0.95, *P* = 0.033). Twenty-two (25.3%) patients experienced acute grade 3 esophagitis and 10 (11.5%) experienced acute grade ≥ 3 radiation pneumonitis. There were 2 (2.6%) late grade 3 pulmonary toxicity and no late grade ≥ 3 esophageal toxicity was observed.

**Materials and Methods:**

A total of 87 patients with locally advanced NSCLC who received SIB-IMRT from Jan. 2009 to Dec. 2012 in our hospital were retrospectively analyzed. Male accounted for 88.5%, with a median age of 61 years old. The SIB-IMRT plans were designed to deliver 50.4–64.0 Gy in 28–33 fractions (1.8–2.1 Gy/fraction) to PTV while simultaneously delivering 60.0–74.3 Gy in 28–33 fractions (2.0–2.5 Gy/fraction) to PGTV.

**Conclusions:**

SIB-IMRT, especially with concurrent chemotherapy, appears to be an effective and safe option to treat patients with locally advanced NSCLC. More prospective clinical studies should be warranted.

## INTRODUCTION

About 30–40% cases of non-small cell lung cancer (NSCLC) belong to the locally advanced stage, which is not suitable for surgical resection. Radiation therapy, usually in combination with chemotherapy is the main treatment for these cases [1]. Prospective studies have shown that the two-year survival rate of stage III NSCLC after chemoradiation was between 22 to 33% [2]. Meanwhile, an improvement of radiation dose could significantly improve the local control and overall survival [3–5]. However, in order to avoid radioactive toxicity to normal tissue surrounded (e.g. radiation pneumonitis and esophagitis etc.), improving of target dose during conventional radiotherapy or three-dimensional conformal radiotherapy (3D-CRT) was usually not easy. Currently, some studies have suggested that the application of intensity-modulated radiotherapy (IMRT) in NSCLC treatment was superior to 3D-CRT in many ways, particularly in reducing the volume dose of normal tissue within radiation range while potentially improving the radiation dose that tumor delivered [6–8]. Yet in these studies regarding IMRT, the radiation dose targeting gross tumor volume and elective regional lymph nodes regions were the same, while technically the former should be higher than the dose delivered to subclinical volume. Simultaneous integrated boost intensity-modulated radiotherapy (SIB- IMRT) could simultaneously deliver a higher dose to the primary tumor volume and a relatively lower dose to the subclinical volume or elective regional lymph nodes regions. However, outcomes and safety for SIB-IMRT in locally advanced NSCLC remains unclear. In recent years, we have treated 87 cases of locally advanced NSCLC with SIB-IMRT technique, and now we perform a retrospective analysis to investigate the efficacy and safety of SIB-IMRT on locally advanced NSCLC.

## RESULTS

### Patients characteristics

A total of 87 patients enrolled in the study. Male accounted for 88.5%, with a median age of 61 years old. The accompanied diseases before diagnosis included: 13 (14.9%) of chronic obstructive pulmonary disease, 4 (4.6%) of coronary heart disease, 5 (5.7%) of diabetes, 18 of hypertension (20.7%), 2 (2.3%) of renal inadequacy, and 1(1.1%) of lower extremity thrombosis. Twenty-four (27.6%) patients received concurrent chemoradiation (CCRT), while the remaining 63 (72.4%) received sequential radiotherapy and chemotherapy or radiotherapy alone. The detailed clinical characteristics of 87 patients were summarized in Table 1.

**Table 1:** Demographics and clinicopathological characteristics of 87 patients

Factors	Number of cases	Percentage (%)
Gender		
Male	77	88.5
Female	10	11.5
Age (years)		
Median (range)	61 (35–85)	
Smoking history		
Yes	72	82.8
No	15	17.2
Clinical staging		
III A	52	59.8
III B	35	40.2
Pathological type		
AC	22	25.3
SCC	52	59.8
NSCC NOS	13	14.9
Pathological grading		
Well-differentiated	4	4.6
Moderately differentiated	24	27.6
Poorly differentiated	37	42.5
Undifferentiated	3	3.4
Unknown	19	21.8
Primary tumor type		
Central	62	71.3
Peripheral	25	28.7
T stage		
T1	8	9.2
T2	41	47.1
T3	20	23.0
T4	18	20.7
N stage		
N0	5	5.7
N1	4	4.6
N2	51	58.6
N3	27	31.0
CCRT		
Yes	24	27.6
No	63	72.4
IGTV volume (cm^3^)		
Median (range)	185.9 (61.7–593.2)	
PTV volume (cm^3^)		
Median (range)	564.8 (183.8–1289.4)	

Abbreviations: AC, adenocarcinoma; SCC, squamous cell carcinoma; NSCC-NOS, non-small cell carcinoma-not otherwise specified; CCRT, concurrent chemoradiation; IGTV, internal gross tumor volume; PTV, planning tumor volume.

### Radiotherapy completion

All patients completed the prescribed radiotherapy. Most of patients (55 cases, 63.2%) received 50.4Gy (in 28 fractions of 1.8Gy/fraction) to planning target volume (PTV) and 64.4Gy (in 28 fractions at 2.3Gy/fraction) to the planning gross target volume (PGTV). Twenty-six (30.0%) patients received 56Gy (in 28 fractions of 2.0Gy/fraction) to PTV and 61.6Gy (in 28 fractions at 2.2 Gy/fraction) to the PGTV. Another 5 (5.7%) received 54.0 Gy (in 30 fractions of 1.8 Gy/fraction) to PTV and 60.0 Gy (in 30 fractions at 2.0 Gy/fraction) to the PGTV. One (1.1%) patient received 64.0 Gy (in 33 fractions at 1.94 Gy/fraction) to PTV and 74.3 Gy (in 33 fractions at 2.25 Gy/fraction) to the PGTV. The median mean lung dose (MLD) was 14.6 Gy (range 11.5–16.9 Gy), V20 was 24.7% (range 19.9–31.8%); esophageal V50, 37.1 % (range 23.3–48.9%); median mean heart dose, 25.4 Gy (range 2.6–28.8 Gy); and median spinal cord maximum dose, 37.4 Gy (range 28.6–45.2 Gy).

### Response evaluation

One month after the completion of radiotherapy, chest and upper abdomen enhanced computed tomography (CT) was performed for the evaluation of efficacy. Of the 87 patients, overall response rate (ORR) was 57.5%, with 13 (14.9%) of complete response (CR), 37 (42.5%) of partial response (PR), 34 (39.1%) of stable disease (SD) and 3 (3.4%) of progression disease (PD). Efficacy evaluation of different subgroups was shown in Table 2.

**Table 2:** Efficacy evaluation of subgroup NSCLC patients [number of cases (%)]

Subgroup	CR	PR	SD	PD	ORR (%)	*P* value
Clinical staging						
III A	8 (15.4)	21 (40.4)	20 (38.5)	3 (5.8)	55.8	
III B	5 (14.3)	16 (45.7)	14 (40.0)	0 (0.0)	60.0	0.534
Pathological type						
AC	3 (13.6)	8 (36.4)	11 (50.0)	0 (0.0)	50.0	
SCC	6 (11.5)	26 (50.0)	18 (34.6)	2 (3.8)	61.5	
NSC NOS	4 (30.8)	3 (23.1)	5 (38.5)	1 (7.7)	53.9	0.317
CCRT						
Yes	6 (25.0)	10 (41.7)	7 (29.2)	1 (4.2)	66.7	
No	7 (11.1)	27 (42.9)	27 (42.9)	2 (3.2)	54.0	0.372

Abbreviations: AC, adenocarcinoma; SCC, squamous cell carcinoma; NSCC-NOS, non-small cell carcinoma-not otherwise specified; CCRT, concurrent chemoradiation; CR, complete response; PR, partial response; SD, stable disease; PD, progression disease; ORR, overall response rate.

### Survival

**Figure 1:**
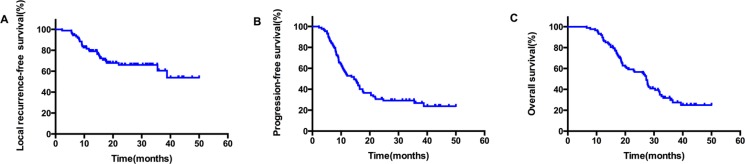
The survival curves in all patients who received SIB-IMRT, local recurrence-free survival (**A**); disease-free survival (**B**); Overall survival (**C**).

**Figure 2:**
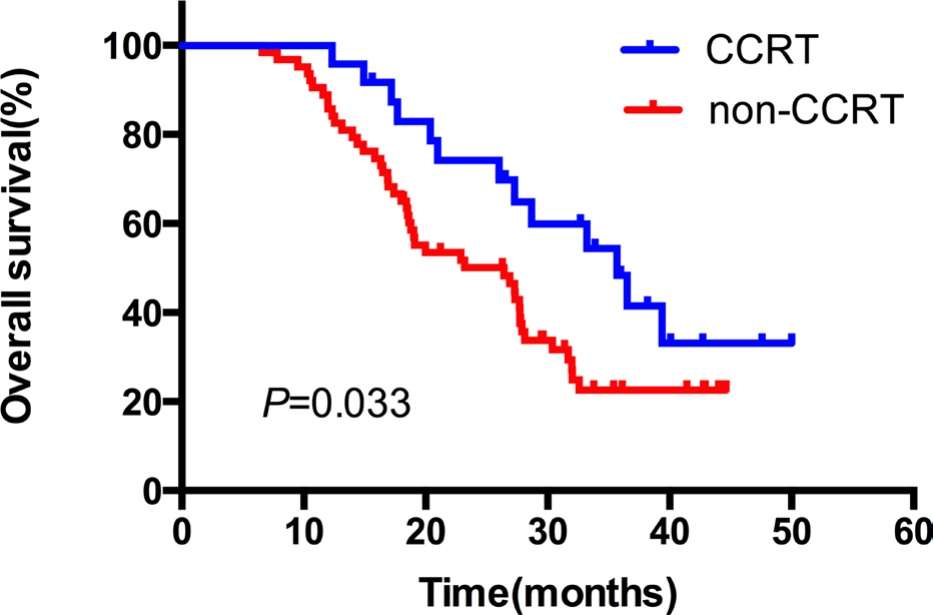
The comparative overall survival curves in patients who received CCRT or not CCRT: concurrent chemoradiation.

**Figure 3:**
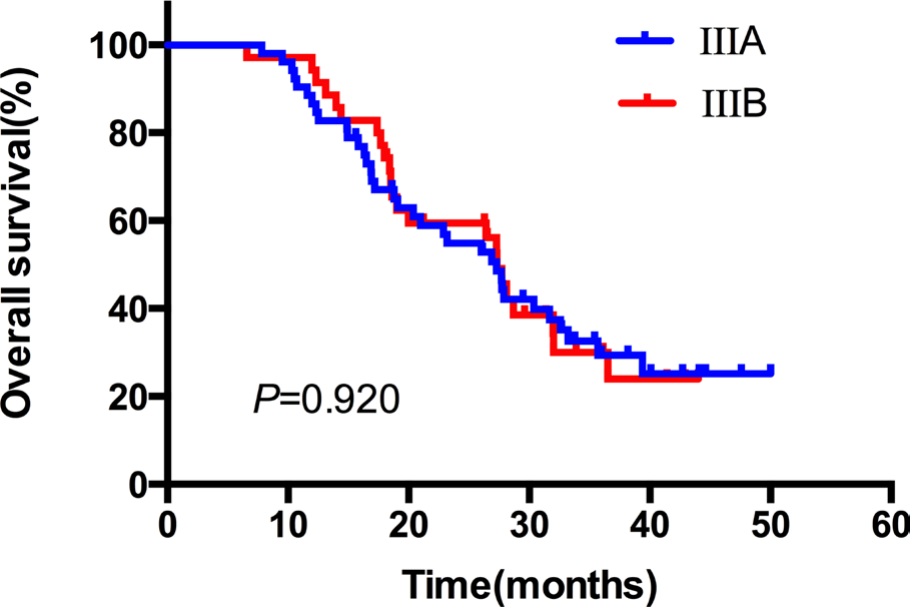
The comparative overall survival curves in patients with different clinical stages after SIB-IMRT

**Figure 4:**
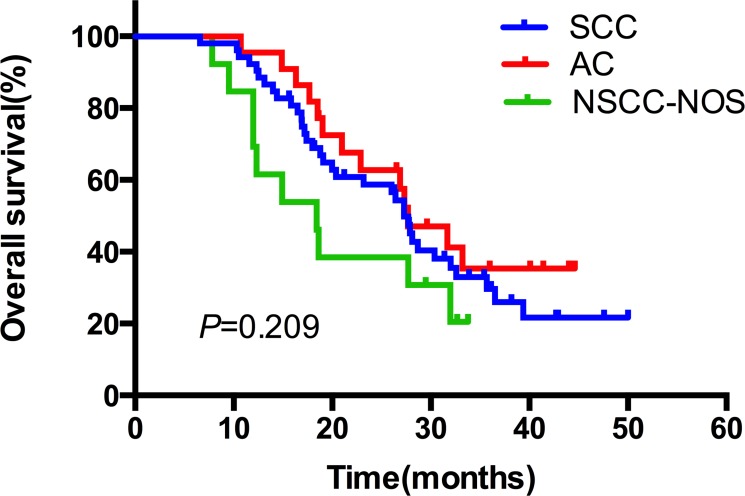
The comparative overall survival curves in patients with different pathological types after SIB-IMRT

The median duration of follow up was 24.6 months, with a range of 3.8–40.5 months. Up to the newest follow-up, 58 in 87 patients have died, 29 surviving. Among all the 58 cases dead, 48 died of local recurrence or distant metastasis, three died of pulmonary infection after treatment, one case of pulmonary hemorrhage, and one died of accident. The other five patients had unknown reason. The one, two, three-year local control rate was 79.0%, 66.1% and 60.5%, respectively (Figure 1A). The one, two, three-year PFS rate was 54.0%, 30.6% and 26.8%, respectively, with a median PFS of 14.5 months (Figure 1B). And the one, two, three-year OS rate was 89.7%, 56.7% and 30.6%, respectively, with a median OS of 27.3 months (Figure 1C). In the subgroup analysis, the median OS in patients who received CCRT was much better than those who did not receive CCRT (35.7 vs. 26.4 months), indicating statistically significant difference between the two subgroups (HR: 0.52, 95% CI: 0.32–0.95, *P* = 0.033) (Figure 2). While in the patients staged III A and III B, the median OS was 27.4 and 27.3 months, and there was no significant difference (HR: 1.03, 95% CI: 0.61–1.74, *P* = 0.920) (Figure 3). Likewise, when it came to pathological subgroups, we found there also was no significant difference among the different pathological types (*P* = 0.209) (Figure 4).

### Toxicity

According to the Radiation Therapy Oncology Group (RTOG) toxicity score criteria, there were 65 (74.7%) patients experienced acute radiation esophagitis, including 22 (25.3%) were scored grade 3 radiation esophagitis and no patient occurred esophagitis above grade 4. Among the 22 patients with grade 3 radiation esophagitis, 16 (18.4%) had received CCRT, and the remaining 6 (6.9%) patients had received sequential chemoradiation. Twenty-three (26.4%) cases were developed acute radiation pneumonitis, including 10 (11.5%) cases were grade 3 or above, with 8 (9.2%) of grade 3 and 2 (2.3%) of grade 4. Among 8 (9.2%) patients who developed grade 3 radiation pneumonitis, 6 (6.9%) were patients who had received CCRT, with the dose of PTV 50.4–64.0 Gy, total lung V20 of 28.2–31.4% and MLD of 14.3–16.7 Gy. And the two (2.3%) patients of grade 4 acute radiation pneumonitis both occurred in stage IIIB patients who had suffered from severe ventilation abnormality before treatment (one case treated by consolidation chemotherapy after radiotherapy, the other treated by radiotherapy alone); and their dose of PTV was 60.0 Gy and 64.0 Gy, lung V20 was 27.6% and 30.7%, and MLD was 15.4, 16.2 Gy, respectively. Late grade 3 pulmonary toxicity occurred in two (2.3%) patients after one year of follow up, while no late esophagitis of grade 3 or above was observed. The acute and late toxicities in lung and esophagus were presented in detail (Table 3).

**Table 3:** Acute and late radiation toxicities

	Acute toxicity [n (%)]				Late toxicity [n (%)]			
**Grade 1**	**Grade 2**	**Grade 3**	**Grade 4**	**Grade 1**	**Grade 2**	**Grade 3**	**Grade 4**
Esophagitis	12 (24.1)	31 (35.6)	22 (25.3)	0 (0.0)	28 (32.2)	11 (12.6)	0 (0.0)	0 (0.0)
Pneumonitis	3 (3.4)	10 (11.5)	8 (9.2)	2 (2.3)	9 (10.3)	6 (6.9)	2 (2.3)	0 (0.0)

### Failure patterns

The patterns of failure are detailed in Table 4. Of all 87 patients, a total of 63 (72.4%) cases had disease progression, among which 28 (32.2%) relapsed within the radiation field and seven (8.0%) in the lymph node outside the radiation field. Another 28 patients developed distant metastasis, including 6 (6.9%) intrapulmonary metastasis, 9 (10.3%) brain metastasis, 6 (6.9%) bone metastasis, 3 (3.4%) liver metastasis, 2 (2.3%) pleural metastasis, 2 (2.3%) adrenal metastasis.

**Table 4:** Failure patterns

	*N*	%
Any failure	63	72.4
Locoregional failure		
PGTV failure	22	25.3
PTV failure	6	6.9
Out-field failure	7	8.0
Distant metastasis	28	32.2
Alive with failure	15	17.2
Alive with no failure	14	16.1
Death from treatment failure	48	55.2
Treatment-related death	3	3.4
Any death	58	66.7

## DISCUSSION

For locally advanced NSCLC patients not suitable for completed resection, the local control and OS after radiotherapy or combined chemoradiation remains unsatisfactory. From early studies, the clinical control rate of NSCLC after conventional radiotherapy was about 50–70%. However, when follow-up with fiberoptic bronchoscopy was used to evaluate the local control rate of NSCLC, it was found that the one-year local control rate of primary lung cancer was less than 20% [9]. The lack of local radiation dose in the primary tumor zone may be the main cause of failure for conventional radiotherapy. Kong [4] indicated that there was obvious dose-effect relationship in the radiation therapy of lung cancer, and that radiation dose escalation could directly improve local control rate. Therefore, to improve the local control rate and long-term survival even quality of life, radiation dose escalation was required. In our present study, we used the SIB-IMRT technology and the results showed a relatively better local control and median survival compared with previous published data [1, 10]. Although only 27.6% patients received CCRT, the one, two, three-year local control rate was 79.0%, 66.1% and 60.5%, respectively. We speculated that the relatively higher dose delivered to the gross tumor benefited the better local control, even OS. It should to be verified in the future perspective studies.

It has been reported that comparing with conventional IMRT technique, SIB-IMRT could increase GVT dosage by 22% with the same radiation dose on the surrounding normal tissue [11]. In our study, PGTV was delivered the total dose of 60.0–74.3 Gy with single dose of 2.0–2.3 Gy, five days per week. The median total dose was 64.4 Gy (in 28 fractions at 2.3 Gy/fraction). Previous study indicated that there was a positive association between radiation dose escalation and local control. When the tumor was delivered escalating dose of 63–69, 74–84 and 92–103 Gy, the control rates were 12%, 35% and 49%, respectively [4]. RTOG 0617 trial was a phase III study comparing a dose of 60 Gy versus 74 Gy delivered concurrently with weekly paclitaxel and carboplatin ± antibody against the epidermal growth factor receptor cetuximab in patients with locally advanced NSCLC. The results surprisingly showed that there was no OS improvement in the group randomized to the higher radiation dose. In fact, the survival in the high-dose arm was worse [12]. Although the actual reasons for the unexpected results remains unclear, a competing explanation is that 74 Gy is too toxic when delivered by the photon-based radiation technologies used in current setting of RTOG 0617. Actually, 74 Gy may still be an inadequate dose for eliminating the tumor, just like the need for biological equivalent dose (BED) above of 100 Gy in early-stage NSCLC. Thus, safely escalating the radiation doses to the gross targets, while sparing the doses to the normal tissue surrounded may be critical to improving the outcome in NSCLC.

In the present study, a total of 82 patients received radiation with PGTV total dose of more than 6160cGy and single dose of more than 220cGy (BED was 62.63 Gy) (α/β = 10). Our long-term results showed that the one, two, three-year PFS rate was 54.0%, 30.6% and 26.8%, respectively. The one, two, three-year OS rate was 89.7%, 56.7% and 30.6%, respectively. The outcome made an improvement to OS and PFS compared with previous literature [1, 2, 13, 14]. Meanwhile, in the subgroup analysis, we found that OS in patients treated with CCRT was significantly better than those who did not receive CCRT. On the other hand, we found that the incidence and severity of treatment-related toxicity, particularly late toxicity, was lower in our study than the previous reports [15, 16], and this was probably due to the following reasons: Prescription dose of PTV in our study was lower than in other studies (the median dose was 5040cGy, only enough for the irradiation of subclinical lesions and elective lymph nodes); Our study had more strict limit of the radiation on normal lung tissue, with lung V20 < 28% for most patients, MLD < 17 Gy; proportion of the patients receiving standard CCRT was relatively low, for quite a few patients received sequential chemoradiation or radiotherapy alone instead, so that patient tolerance was better. As a combination of the above, we may conjecture that treatment intensity in our study could be increased still. So in our further research, standard CCRT or more radiation dose to PGTV should be applied under the premise of maintaining the intensity of radiotherapy, in order to achieve better local control and survival.

In terms of failure patterns, our results suggested most of the patients relapsed within the radiation field or distant metastasis. Only seven (8.0%) relapsed in the lymph node outside the radiation field, indicating that the application of SIB-IMRT irradiation technique in the treatment of locally advanced NSCLC would not increase the recurrence rate of elective lymph node regions.

The present study had some limitation. Firstly, we did not use four-dimensional CT simulation, which could delineate more precise PGTV considering the tumor respiratory movement. Secondly, the size of enrolled population was small, which may cause the selection bias. Meanwhile, the treatment strategies of the patients were not uniform. Last but not least, positron emission tomography scan was rarely used before the treatment and during the period of follow-up, which means that some metastatic sites may have been missed and the location of failure in relation to the PGTV and PTV could not be accurately identified.

In conclusion, SIB-IMRT, especially with concurrent chemotherapy, appears to be an effective and safe option to treat patients with locally advanced NSCLC. However, our study was retrospective and only involved a few cases. The conclusion was still to be confirmed by further randomized controlled studies with larger sample sizes.

## MATERIALS AND METHODS

### Patients selection

All 87 cases involved were pathologically proved NSCLC patients in Zhejiang Cancer Hospital from January 2009 to December 2012. Inclusion criteria were as follows: age ≥ 18 years; newly diagnosed cases of primary lung cancer; confirmed unsuitable to receive radical resection by surgeons or patient refusal; stage IIIA or IIIB according to the 7th edition of the American Joint Committee on Cancer (AJCC) staging manual for lung cancer; Eastern Cooperative Oncology Group (ECOG) performance status (PS) score of 0–2; Forced expiratory volume in the first second (FEV1) ≥ 1000 cm^3^, weight loss of less than 5% in the last three months; with measurable lesions by the response evaluation criteria in solid tumors (RECIST) 1.1 criteria; without other serious cardiopulmonary, hepatic or myeloid comorbidities. The study was approved by our hospital's review board. All participants gave written informed consent.

### Radiotherapy

**Figure 5:**
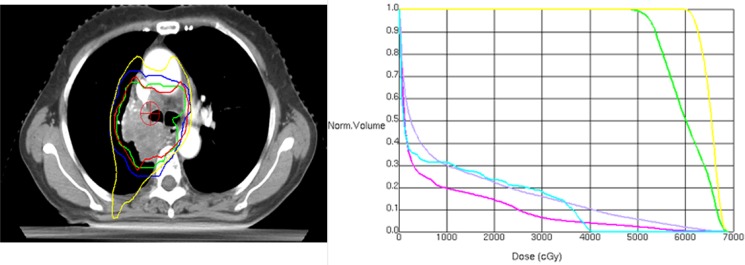
Isodose curves (left) and dose-volume histograms (right) of a simultaneously integrated boost intensity-modulated radiotherapy plan for a patients with locally advanced NSCLC (Left: green line represents the internal gross target volume (IGTV), blue line represents the planning target volume (PTV); Right: the yellow line outlines IGTV (61.6 Gy/28 fractions), and the green line area includes the PTV (50.4 Gy/28 fractions), red for heart, blue for spinal cord, gray for lung).

All patients took supine position and were fixed with thermoplastic mask, with arms crossed above the head. Enhanced CT scan was performed with large diameter spiral CT simulator (GE LightSpeed RT, USA) in quiet breathing conditions. Scanning range was from the upper edge of the second cervical vertebra to the lower edge of the second lumbar, and the thickness was 5 mm. The scanned were transmitted to the Philips Pinnacle 9.2 three-dimensional treatment planning system (Philips Medical Systems, Bothell, WA). The gross target volume (GTV) was defined as any visible primary lesions, and all lymph nodes with a diameter > 1 cm in short axis were included. The primary lesions were delineated in lung window (window width 850 Hu, window level **−**750 Hu) and positive lymph nodes and normal organs were determined in mediastinal window (window width 400Hu, window level 20 Hu). The clinical target volume (CTV) was generated by expanding GTV a 6–8 mm margin and the high-risk lymph nodal regions and the ipsilateral hilar without extending into uninvolved organs. Another 0.6–1.0cm margin was expanded to generate the PTV. PGTV was generated with an extension margin of 0.4–0.6 cm in the anterior-posterior (A-P) direction and left-right (L-R) direction, and 0.8–1.0 cm in the superior lobes, 1.0–1.5cm in the mid or lower lobes in the cranial-caudal (C-C) direction. Prescription dose was defined as 95% of the receiving dose of PTV and PGTV, with the difference of internal target dose uniformity < 5%, and internal target maximum dose point ≤ 110%. Lung V20 (percentage of the total lung volume receiving 20 Gy or more radiation dose) was generally required to be less than 28% and was changed appropriately (not exceeding 34%) according to patient's physical conditions and comprehensive treatment model. MLD < 17%, maximum spinal cord dos < 45 Gy; esophageal V50 < 50%, heart V40 < 50% were other demands to meet. Radiation was performed with linear accelerator 6MV-X. Of all 87 patients, prescription dose was PTV 50.4–64.0 Gy/28–33F, single dose 1.8–2.0 Gy; PGTV was 60.0–74.3 Gy/28–33F, with single dose of 2.0–2.3 Gy. The example of SIB-IMRT treatment plan was shown in Figure 5.

### Chemotherapy and other therapy

Among all 87 patients, 74 (85.1%) patients administrated platinum-based chemotherapy, of which 24 (27.6%) were CCRT and 50 (57.5%) were sequential chemotherapy. Twenty-one patients received vinorelbine (25 mg/m2 intravenously, Days 1,8) and cisplatin (25 mg/m2 intravenously, Days 1–3); 28 cases of gemcitabine (1000 mg/m2 intravenously, Days 1,8) and cisplatin (25 mg/m2 intravenously, Days 1–3); 22 cases of taxane-based (paclitaxel 135 mg/m2 or docetaxel 25 mg/m2 intravenously, Day 1) chemotherapy combined with cisplatin (25 mg/m2 intravenously, Days 1–3), and another three cases of etoposide (50 mg/m2 intravenously, Days 1–5) and cisplatin (50 mg/m2 intravenously, Days 1, 8) (all concurrent with radiotherapy). The number of chemotherapy cycles was ranged from one to six, with a median of four cycles. During treatment, seven of the 87 patients were given sodium glycididazole for synchronization sensitizing, and nine patients were given amifostine to avoid radioactive lung and esophageal injury. Two patients received concurrent thermotherapy and one received target therapy by taking erlotinib.

### Follow-up and efficacy evaluation

The treatment response was evaluated within three months after radiotherapy referring to the Response Evaluation Criteria in Solid Tumors (version 1.1) [17]. After the first evaluation, follow-up should be performed every 3 months over the first 2 years and every 6 months thereafter. The medical test included physical examination, complete blood count, chest and upper abdomen CT, brain magnetic resonance imaging or CT scan, and bone scan (if necessary). On the other hand, the evaluation of toxicity in esophagus and lung was made according to the RTOG radiation injury grading standards.

### Statistical analysis

We collected clinical and pathological data mainly on the following patient characteristics: gender, age, smoking history, clinical stage, pathological type, grading, primary tumor location, chemotherapy and target volume. A nonparametric Mann–Whitney *U* test was used for comparisons among the efficacy of the groups. Failure patterns were defined as the first site of failure. Local recurrence included the primary tumor and regional lymph nodes. Survival duration was calculated from the first day of the treatment to that of the first occurrence of the considered event (local recurrence, disease progression, or death) or last follow-up time. Overall survival (OS), local recurrence-free survival (LRFS) and progression-free survival (PFS) rates were calculated by the Kaplan–Meier method and curves compared with log-rank tests. Cox proportional hazard models were used for the calculation of hazard ratio (HR) of recurrence or death. *P* < 0.05 was considered to be statistically significant. The reported confidence intervals (CI) are assumed to have a coverage probability of 95%. All the analyses were performed using SPSS 21.0 (Statistical Package for Social Sciences, SPSS Inc., Chicago, IL, USA).
